# Supplementary figures and images for: 4’-Fluorouridine mitigates lethal infection with pandemic human and highly pathogenic avian influenza viruses
Source: PLoS Pathog. 2023 Apr 17;19(4):e1011342. doi: 10.1371/journal.ppat.1011342 (PMC10138230; doi:10.1371/journal.ppat.1011342)

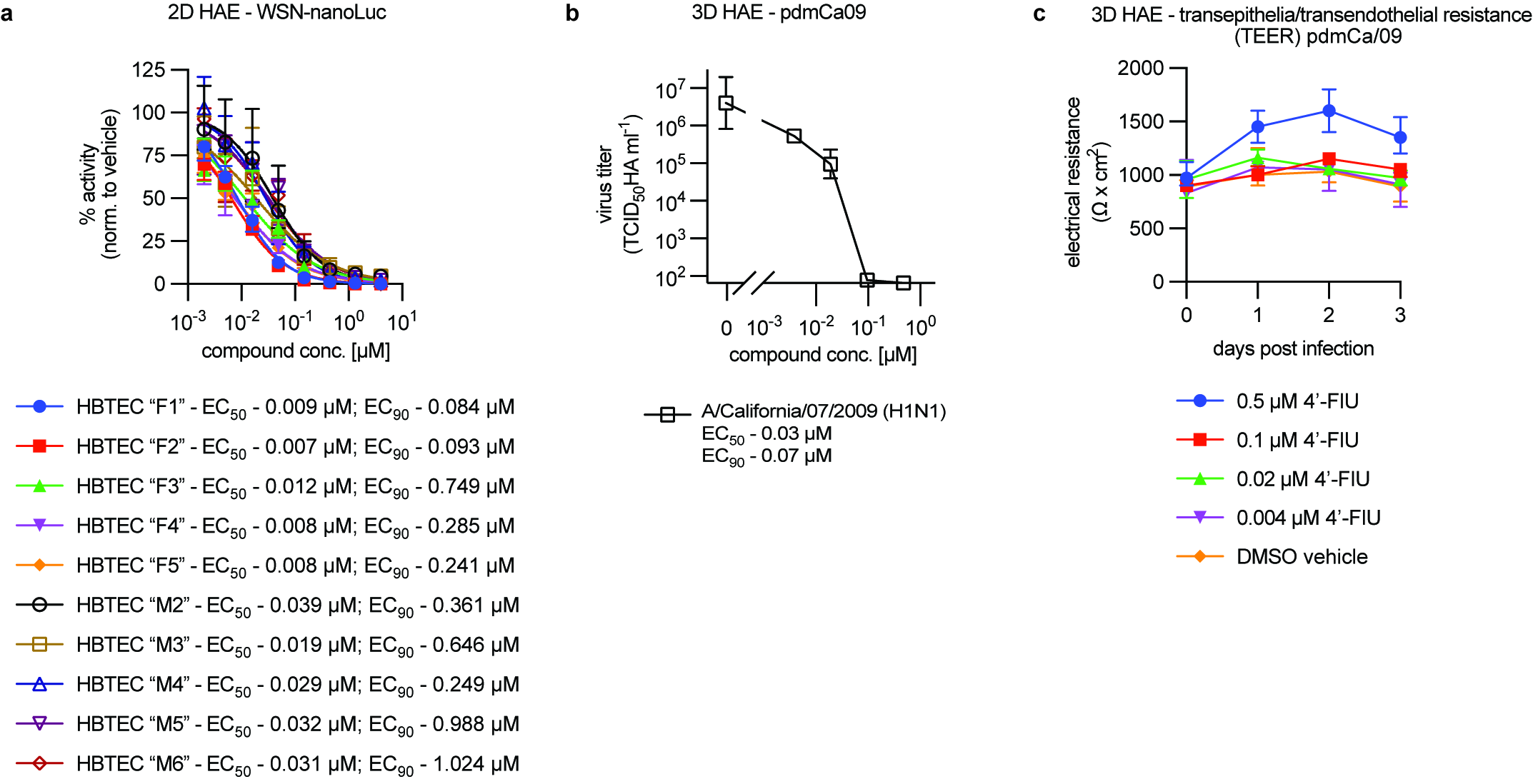

Supplement: S1 Fig — a) 4’-FlU antiviral activity in 2D HAE cultures. Dose-response assays of 4’-FlU against a A/WSN/33 (H1N1) nano-luciferase reporter virus (WSN-nanoLuc) on undifferentiated HAEs derived from 10 different healthy donors, five male and five female. Lines represent 4-parameter variable slope regression models; symbols show data means ± SD; n = 6. EC50 and EC90 concentrations are specified. b) Dose-response assay of 4’-FlU against pdmCa09 on a well-differentiated HAE culture grown at air-liquid interface. Cultures were infected apically, compound was added to the basolateral chamber; line intersects, and symbols show, geometric means ± SD of apically shed virus (n = 3). EC50 and EC90 values based on 4-parameter variable slope regression modeling are shown. c) TEER measurements of HAE cultures from (b). Symbols show individual measurements (individual transwells); lines intersect, and symbols show, data means ± SD. (TIF) [file ppat.1011342.s001.tif]

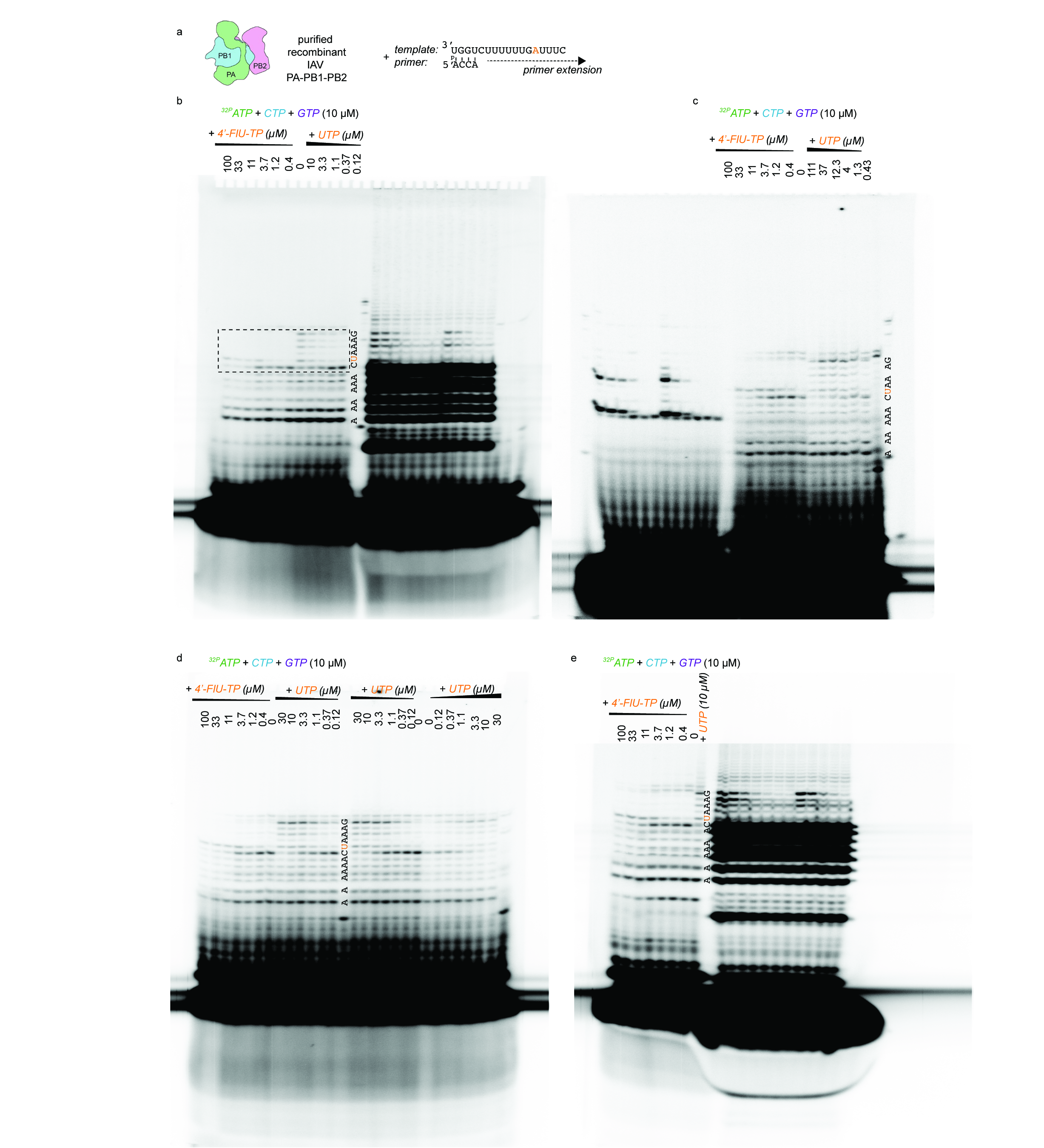

Supplement: S2 Fig — a) Template and primer used in the reaction. b-e) Independent repeats of primer extension assay by IAV polymerase. Dashed rectangle shows insert presented in Fig 1E. Replicates were included in quantitations presented in Fig 1E and 1F. (TIF) [file ppat.1011342.s002.tif]

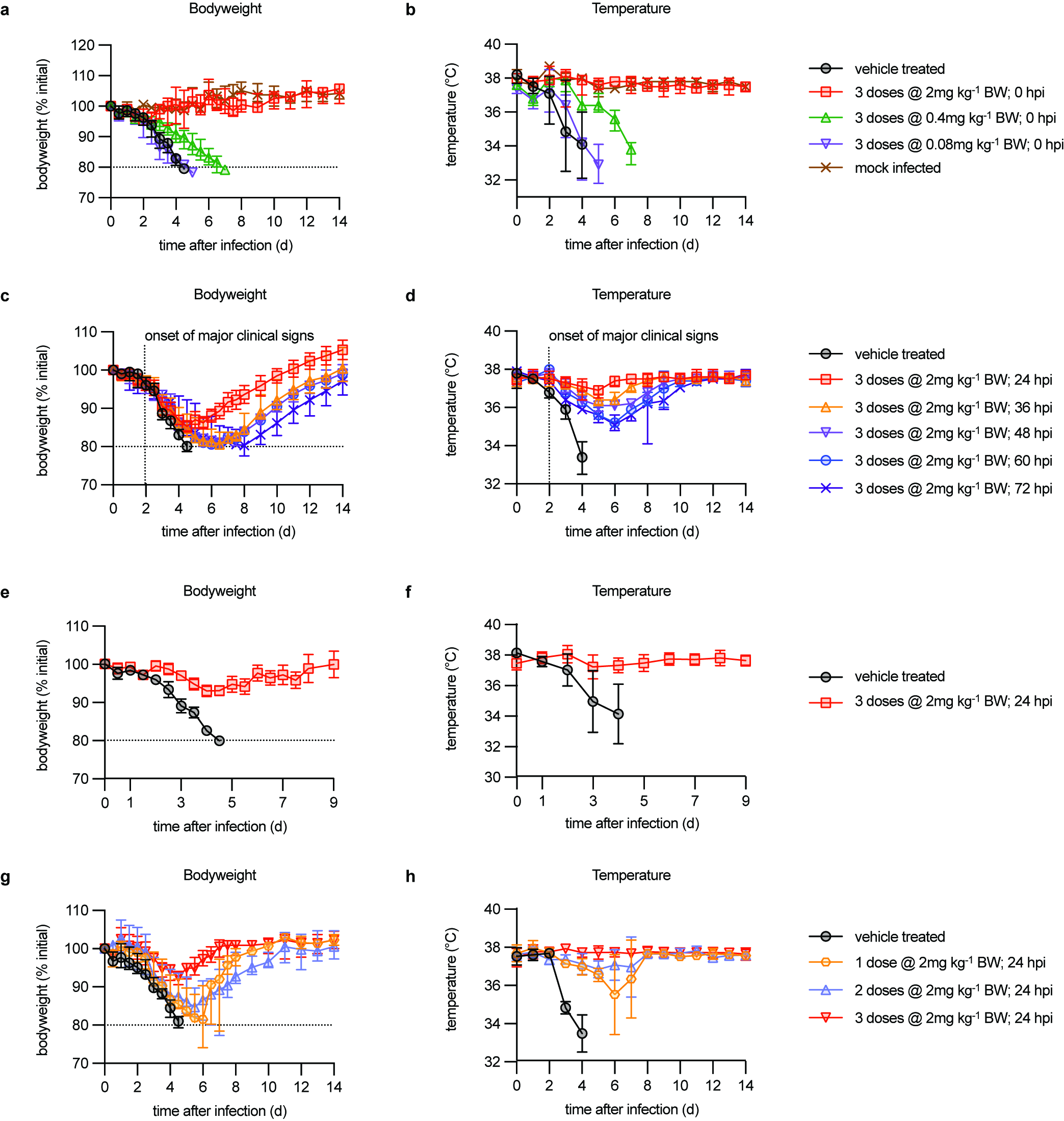

Supplement: S3 Fig — a-h) Body weight measurements taken twice daily (a, c, e, g) and rectal body temperature determined once daily (b, d, f, h). Dashed horizontal line specifies humane endpoint of 20% body weight loss; lines intersect, and symbols show, data means ± SD; n values are specified in main Fig 2. Results are shown for lowest efficacious dose (a-b), latest onset of efficacious treatment (c-d), virus clearance (e-f) and minimal number of doses required (g-h) studies. (TIF) [file ppat.1011342.s003.tif]

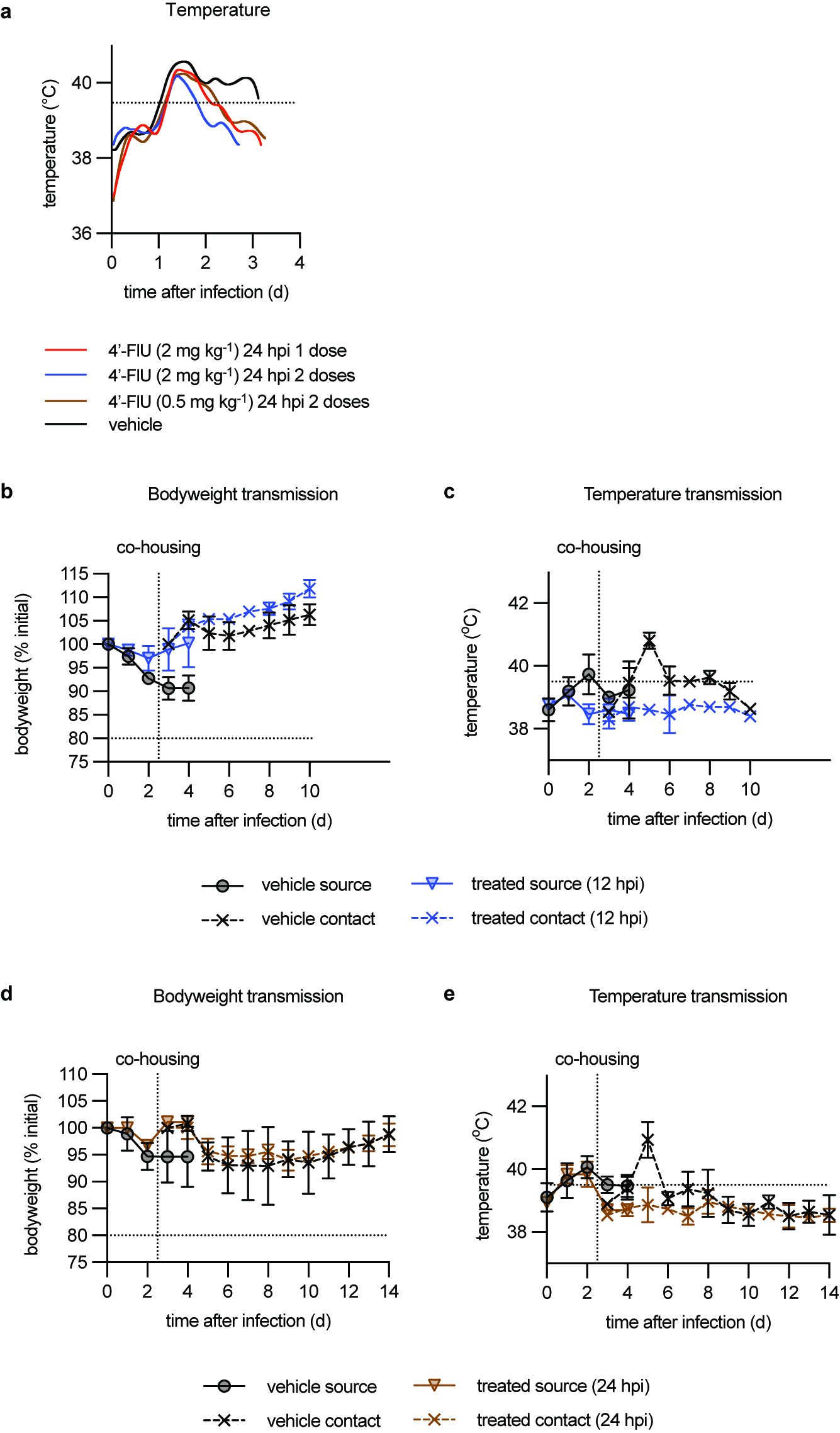

Supplement: S4 Fig — a) Continuous telemetric assessment of body temperature of ferrets in the 4’-FlU efficacy study. b-e) Once-daily body weight (b, d) and rectal body temperature (c, e) of ferrets involved in the pdmCa09 transmission studies. Lines intersect, and symbols show, data means ± SD. Dashed lines in (a,c,e) represent onset of fever; dashed lines in (b,d) specify predefined endpoint. (TIF) [file ppat.1011342.s004.tif]

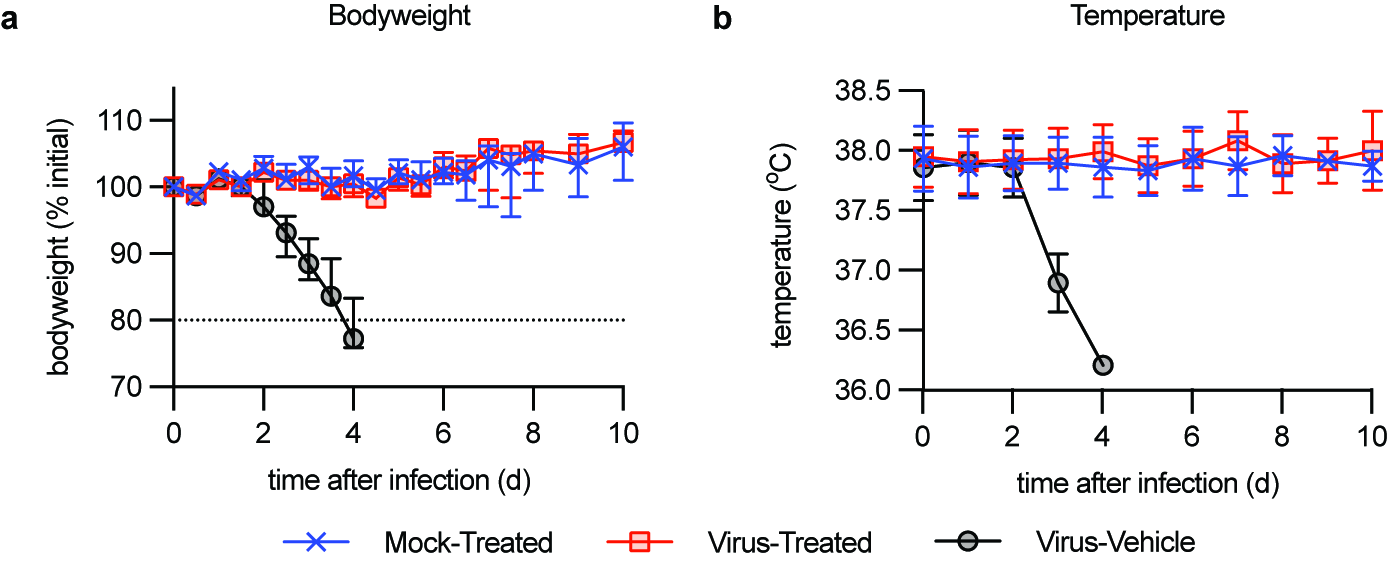

Supplement: S5 Fig — a-b) Body weight measurements taken twice daily (a), and rectal body temperature determined once daily (b). Lines intersect, and symbols show, data means ± SD; n values are specified in Fig 4. Dashed line in (a) specifies predefined endpoint. (TIF) [file ppat.1011342.s005.tif]

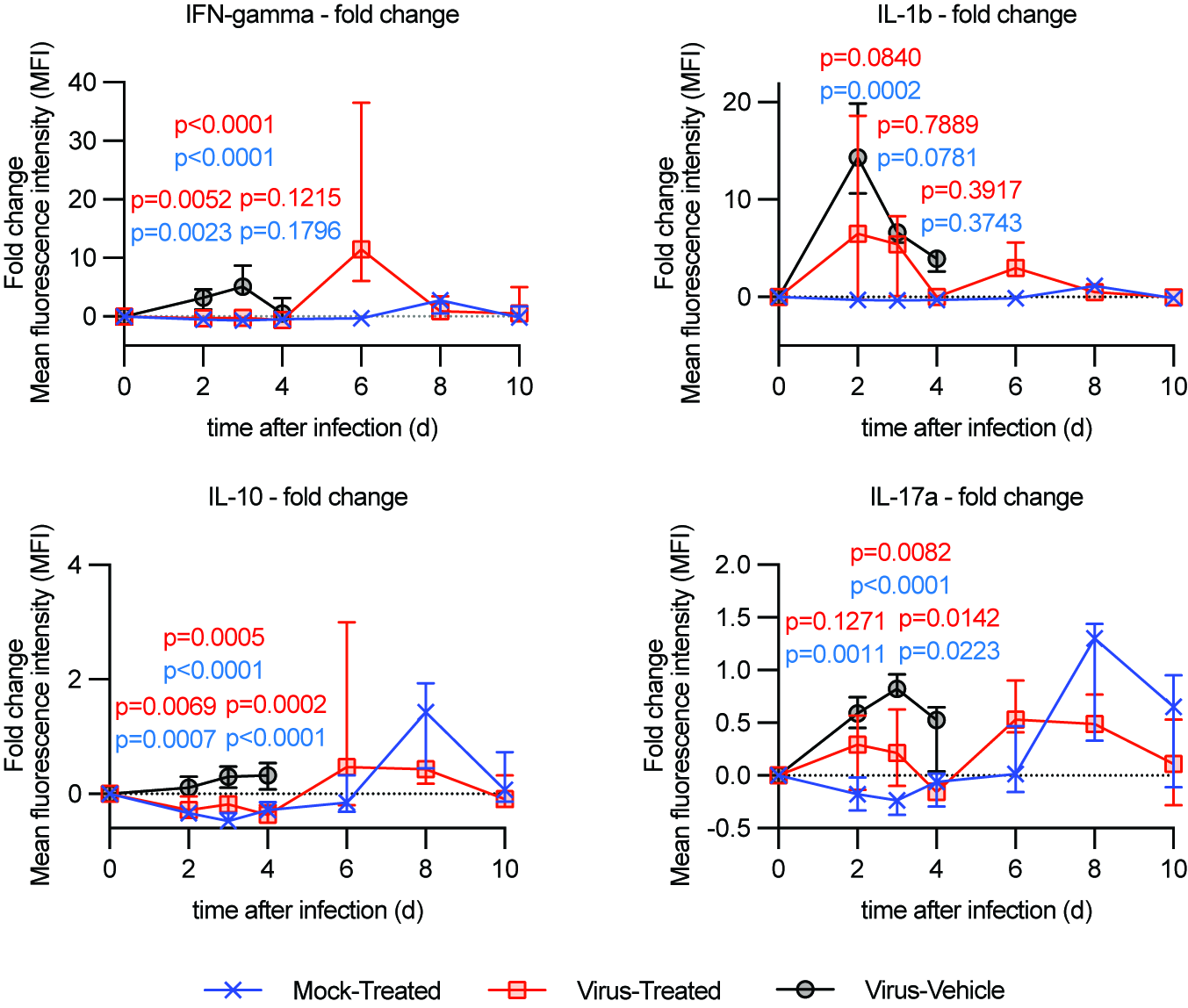

Supplement: S6 Fig — IFNy, IL-1b, IL-10, and IL-17a levels present in BALF of animals treated as in Fig 4A are shown, calculated relative to levels at time of infection. Lines intersect, and symbols show, data medians with 95% CI; 2-way ANOVA with Tukey’s post hoc test; P values are specified. (TIF) [file ppat.1011342.s006.tif]

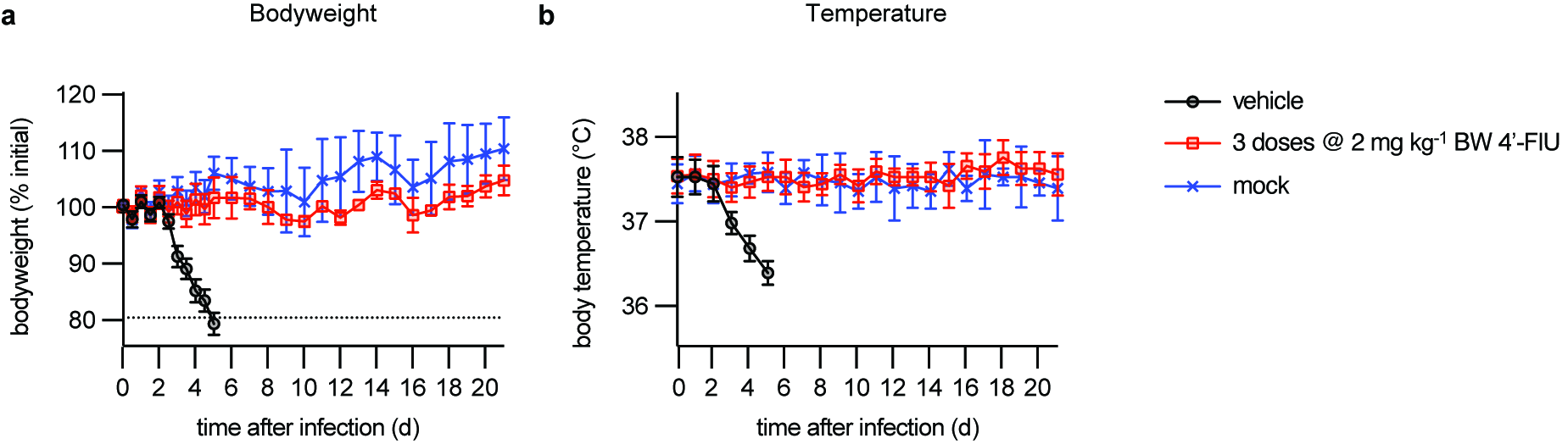

Supplement: S7 Fig — a-b) Body weight measurements taken twice daily (a), and rectal body temperature determined once daily (b). Lines intersect, and symbols show, data means ± SD. Dashed line in (a) specifies predefined endpoint. (TIF) [file ppat.1011342.s007.tif]

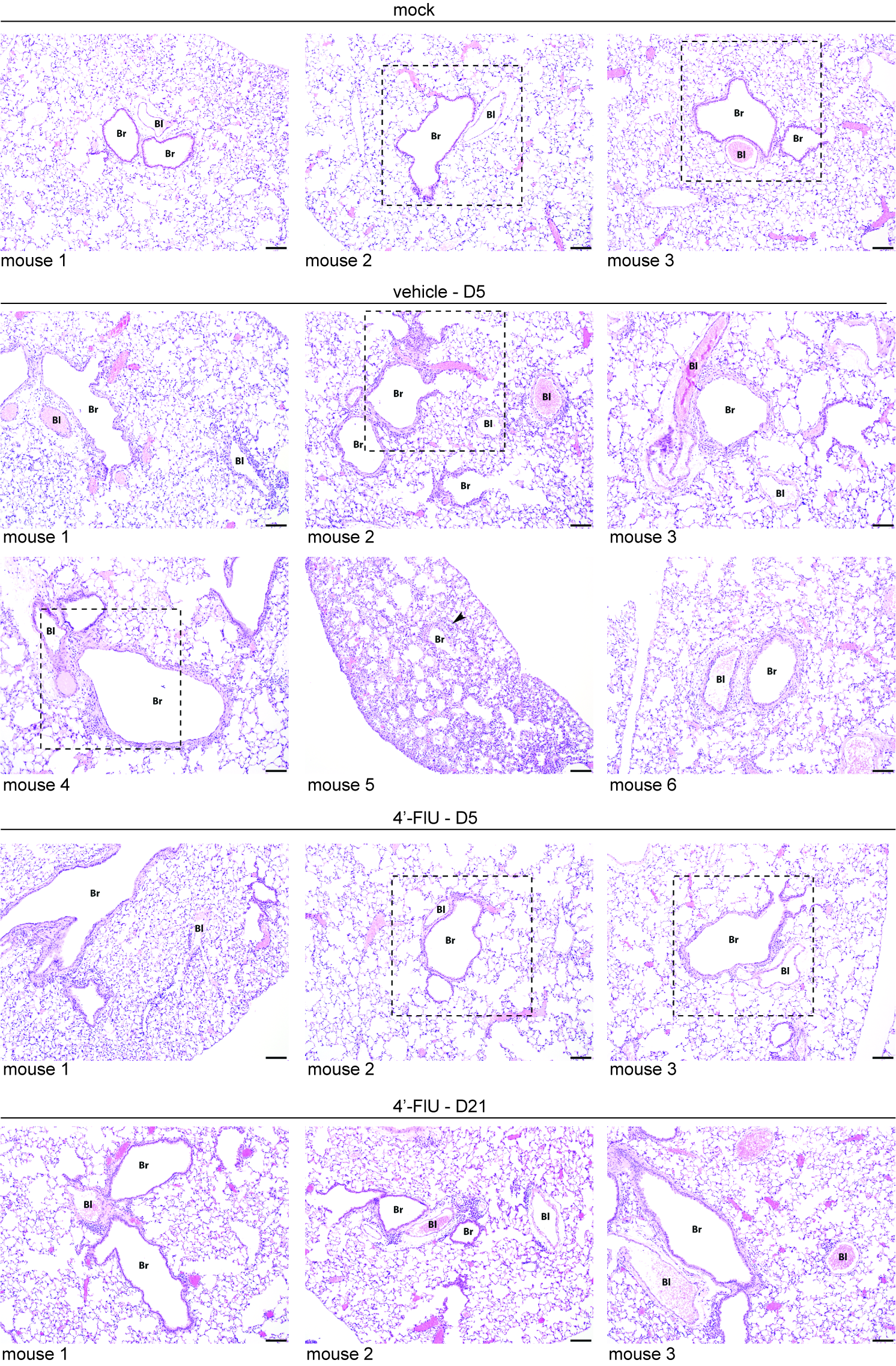

Supplement: S8 Fig — Lungs were extracted on study days 5 or 21 (infected and treated animals only) of all animals examined in this study; tissue section were H&E stained; dashed rectangle shows sections presented in Fig 4E.; magnification 10×; scale bar 100 μm; Br, bronchiole; Bl or arrowhead, blood vessel. (TIF) [file ppat.1011342.s008.tif]

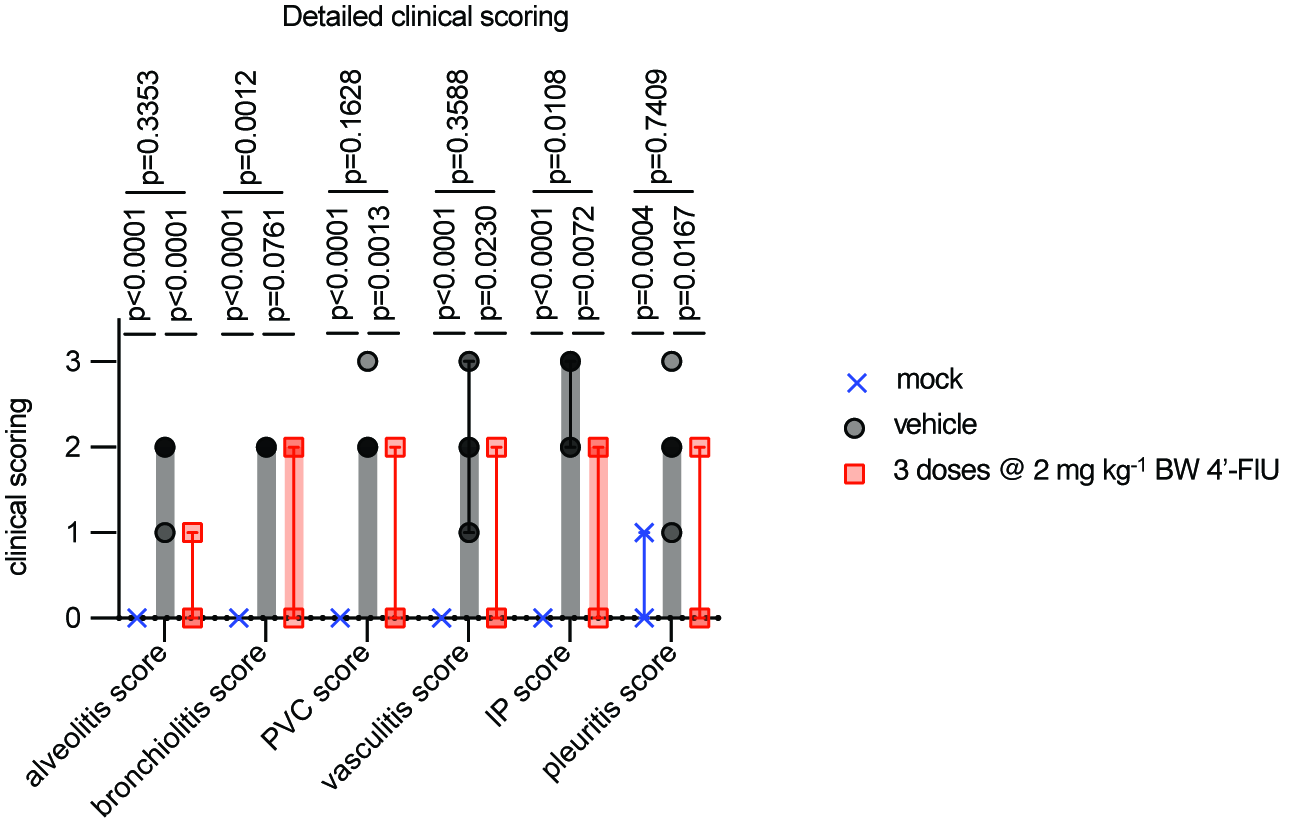

Supplement: S9 Fig — Individual clinical scores for alveolitis, bronchiolitis, perivascular cuffing (PVC), vasculitis, interstitial pneumonia (IP), and pleuritis. Columns show data medians, symbols represent individual animals; 1-way ANOVA with Tukey’s post hoc test; p values are specified; n values are specified in Fig 4F. (TIF) [file ppat.1011342.s009.tif]

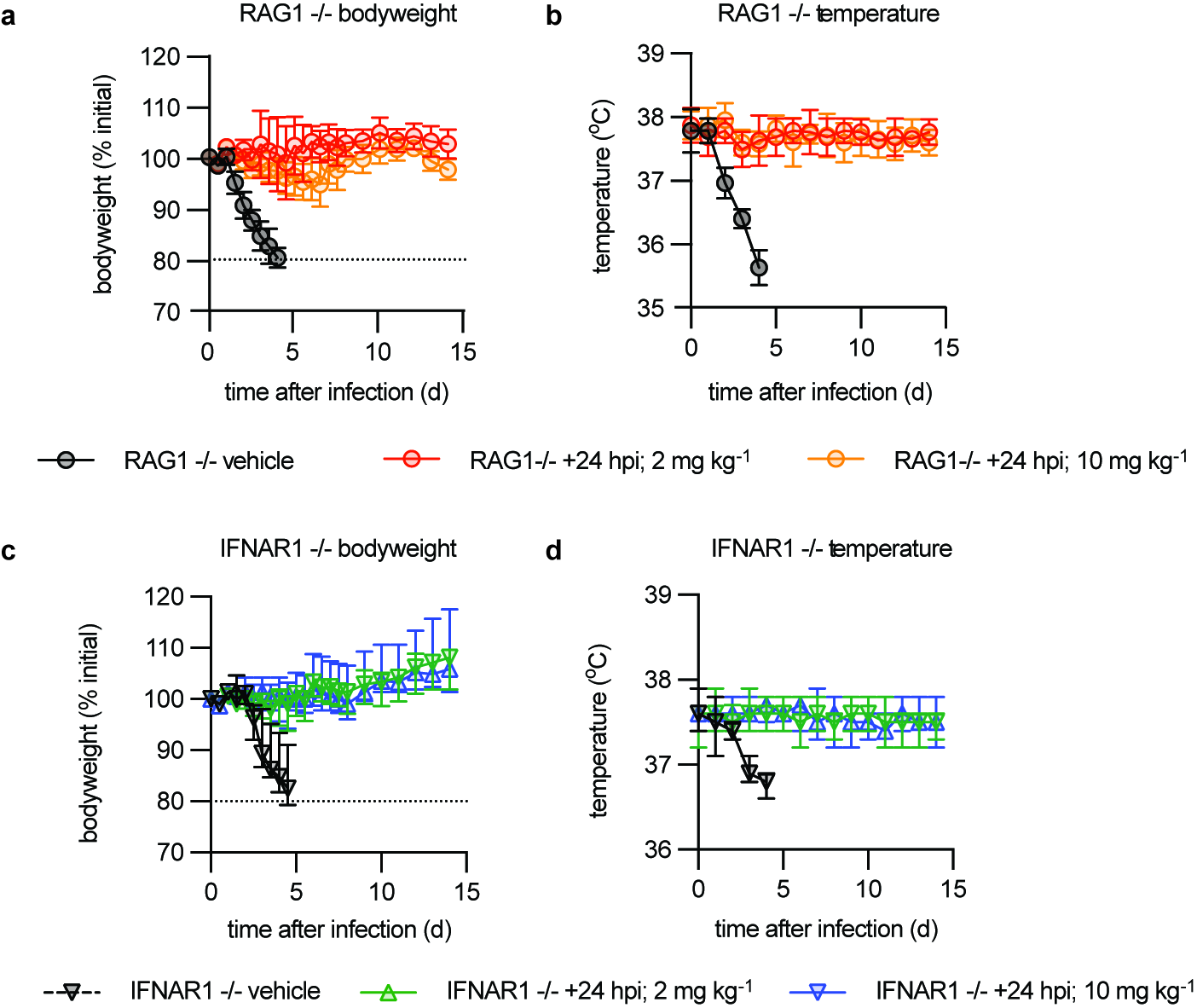

Supplement: S10 Fig — a-c) Shown are body weight measurements taken twice daily (a, c), and rectal body temperature (b, d) determined once daily. Lines intersect, and symbols show, data means ± SD; n values are specified in Fig 5. Results are shown for RAG1 KO (a-b) and IFNAR1 KO (c-d) animals. Dashed lines in (a,c) specify predefined endpoint. (TIF) [file ppat.1011342.s010.tif]

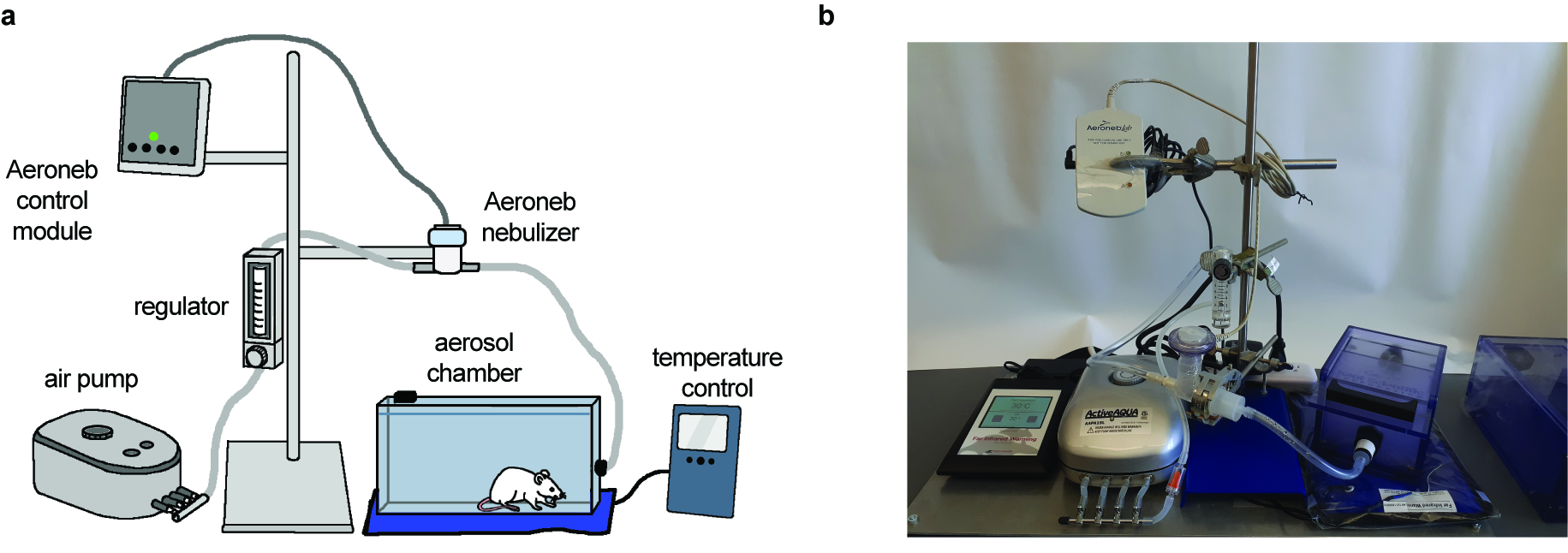

Supplement: S11 Fig — a-b) Schematic representation of the system (a) and photograph of the assembly (b). (TIF) [file ppat.1011342.s011.tif]

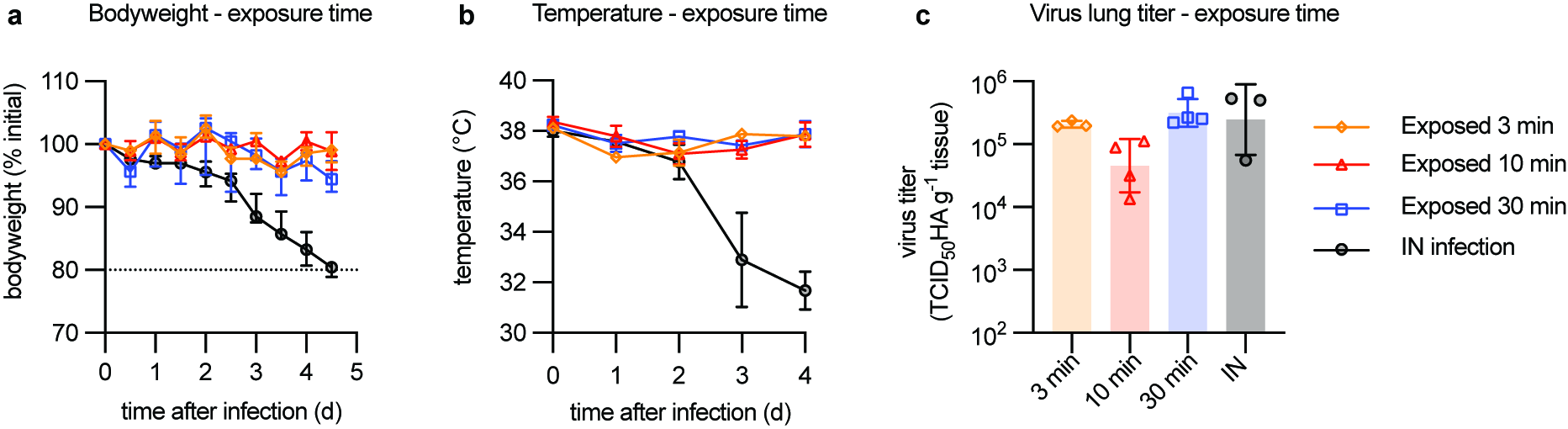

Supplement: S12 Fig — a-b) Body weight measurements taken twice daily (a), and rectal body temperature (b) determined once daily. Lines intersect, and symbols show, data means ± SD. c) Lung viral titers determined 4 days after infection. Columns represent geometric means ± SD; symbols show individual animals (n = 4). Dashed line in (a) specifies predefined endpoint. (TIF) [file ppat.1011342.s012.tif]

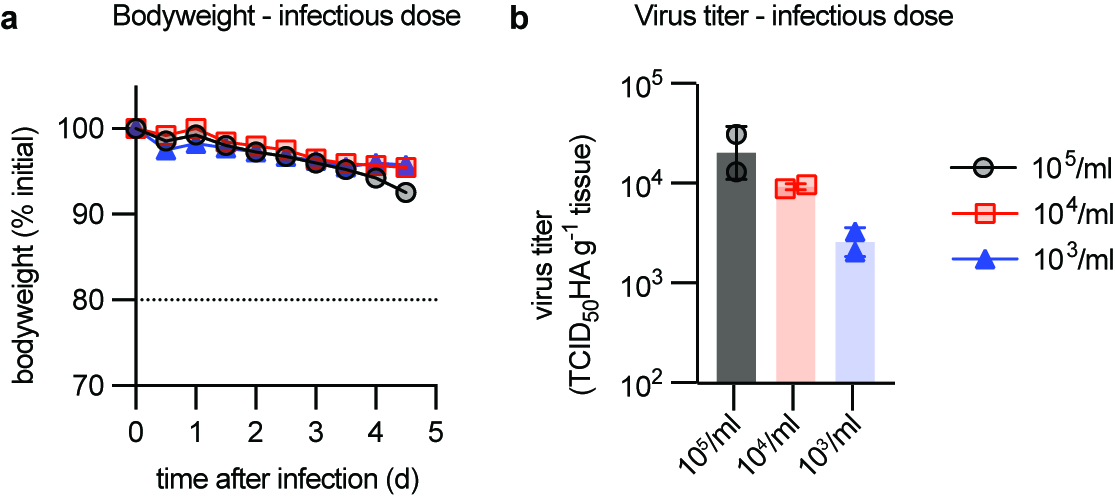

Supplement: S13 Fig — a) Body weight measurements taken twice daily. Lines intersect, and symbols show, data means. b) Lung viral titers determined 4 days after infection. Columns represent data mean with range; symbols show individual animals (n = 2). Dashed line in (a) specifies predefined endpoint. (TIF) [file ppat.1011342.s013.tif]

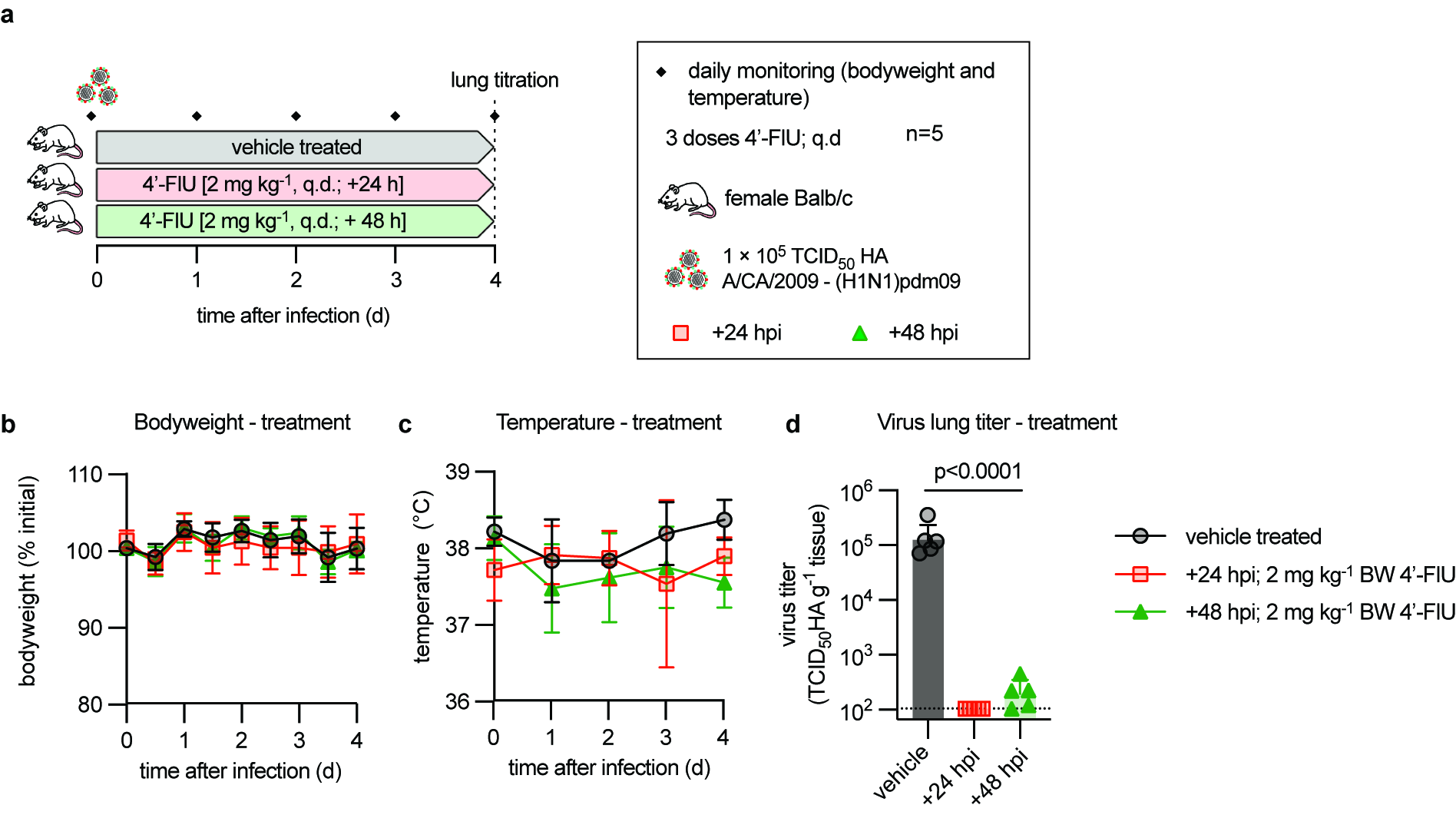

Supplement: S14 Fig — a) Efficacy study schematic; n values are specified. b-c) Body weight measurements of animals from (a) taken twice daily (a), and rectal body temperature (b) determined once daily. Lines in (b-c) intersect, and symbols show, data means ± SD. d) Lung viral titers determined 4 days after infection. Columns represent geometric means ± SD, symbols show individual animals; 1-way ANOVA with Dunnett’s post hoc test; P values are specified. Dashed line in (d) specifies limit of detection. (TIF) [file ppat.1011342.s014.tif]

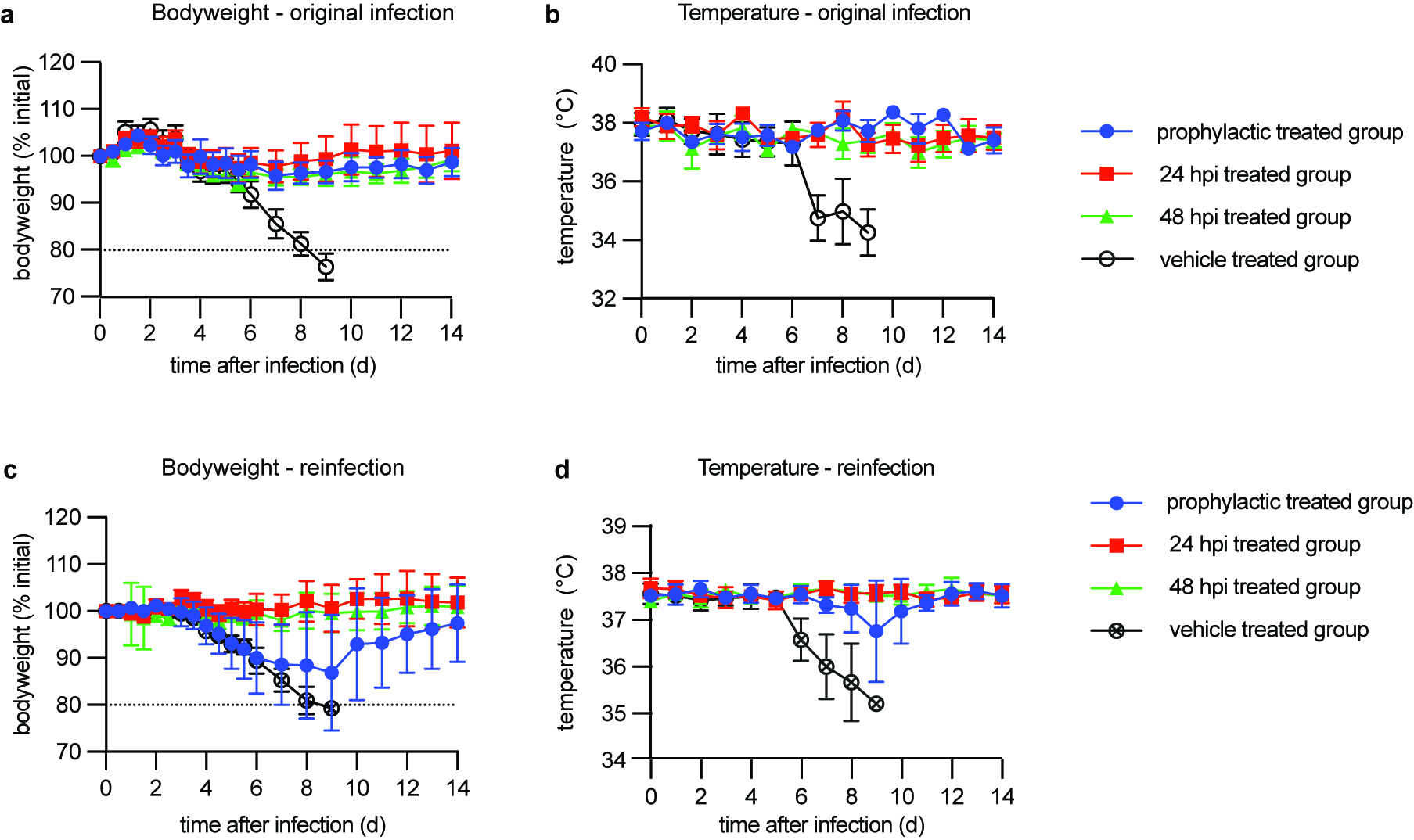

Supplement: S15 Fig — a-d) Body weight measurements taken twice daily (a, c), and rectal body temperature determined once daily (b, d). Lines intersect, and symbols show, data means ± SD. Results are shown for animals after the original infection (a-b) and after homotypic reinfection (c-d). Dashed lines in (a,c) specify predefined endpoint. (TIF) [file ppat.1011342.s015.tif]
