# Supplementary material for: 4’-Fluorouridine mitigates lethal infection with pandemic human and highly pathogenic avian influenza viruses
Source: PLoS Pathog. 2023 Apr 17;19(4):e1011342. doi: 10.1371/journal.ppat.1011342 (PMC10138230; doi:10.1371/journal.ppat.1011342)
Supplement: Attachment — Submitted filename: Response letter.docx [file ppat.1011342.s018.docx]

**Response to Reviewers’ Comments**

**Reviewer #1**

The work presented in this manuscript evaluated the efficacy of the 4’-Fluorouridine derivative compound, which was previously shown to be highly effective against SARS-CoV-2 and RSV, on influenza A and B viruses in vitro and in vivo (with two animal models. The results demonstrate that the compound is highly potent against highly pathogenic influenza viruses including avian flu H5N1. It was shown that the compound is still effective for the mouse survival 60 hours post viral infection, and it can block direct viral transmission using a ferret model. The experiments were carefully designed and performed, and the manuscript well written. This compound has potential to be developed as a new anti-flu drug.

Thank you very much for this assessment of the importance of our work.

1. Add statistics/significance to the survival curves in the figures

Statistics (median survival; Kaplan-Meier simple survival analysis) have been added to figures 2d,f,k, 5b,g, and 6b,d.

2. Is there an explanation for the discrepancy in the potency between the flu isolates in Figure 1B?

This is an excellent question. There is a molecular explanation indeed. Full resistance profiling of this compound against IAVs is ongoing and some mediators of moderate resistance have emerged. Some of these mutations are present in circulating strains such as A/swine/Spain/53207/2004 (H1N1) and A/duck/Alberta/35/76 (H1N1). The moderate sensitivity shift seen in Fig 2b confirms these evolving profiles that were generated through adaptation of pdmCa09 and rebuilding of candidate mutations in recombinant virus for confirmation. As part of this resistance profiling study, we also re-introduced all resistant rec pdmCa09 into mice and noted that treatment with 4’-FlU overcomes the moderate level of resistance achievable, still mediating complete survival. We considered combining both studies in a single paper, but each is a data-rich full-length manuscript, which would make for an overwhelmingly long and dense read when combined. Accordingly, we decided to leave the analysis of the molecular basis for the sensitivity differences for the resistance profiling study, but stated in this revision that “whole genome sequencing of 4’-FlU-experienced virus populations isolated from infected and treated animals showed no allele-dominant divergence from the inoculum population. However, future resistance profiling of 4’-FlU against influenza viruses will be required to fully appreciate the height of the genetic barrier against viral escape from inhibition and possibly explore the molecular basis for the slight virus strain-dependent differences in sensitivity to 4’-FlU that we observed in cell culture dose-response assays” (page 13, lines 318-323).

**Reviewer #2**

The manuscript describes in vitro and in vivo efficacy of 4’-fluorouridine (4FIU) against a number of influenza A virus strains. This drug had previously been shown to have activity against RSV and SARS-CoV-2 by functioning to prematurely terminate RNA synthesis, and this mechanism of action was shown to be even more potent against influenza. The study’s strengths are that it combines a wide range of in vitro assays with a comprehensive analysis of drug efficacy in two animal species, in addition to immunocompromised mice.

Figure 1B – is it surprising that the EC50s of the drug for IBV and the duck and swine derived IAVs is 10-50 fold higher than that seen with the human IAVs tested?

This is not surprising in the light of our ongoing resistance profiling study. Please see our response to comment #2 of reviewer #1 above.

Figure 1C – given the disparate activity of 4FIU against IBV and swine/duck IAV, it would be important to ensure that the drug was still active in the same dosing range on the HAE organoid cultures.

For this revision, we have tested 4’-FlU against pdmCa09, A/swine/Spain/53207/2004 (H1N1), and representatives of both IBV lineages, B/Memphis/20/1996 (Yamagata) and B/Malaysia/2506/2004 (Victoria) on primary human airway epithelium (HAE) cells and observed consistently active concentrations in the sub-micromolar range. We agree with the reviewer that this is an important piece of information and have replaced the original figure 1c with this new dataset. The original subfigure 1c was relocated to supplementary figure S1b Fig and the text revised accordingly.

HPAI experiments – its not clear why the authors boosted the drug dose to 5 mg/kg in the HPAI experiments, when 2 mg/kg was used in experiments with 2009 H1N1.

We have clarified in this revision (page 11, lines 254-256) that the 2.5-fold higher dose was selected to partially compensate for lower brain tissue exposure of 4’-FlU (shown in figure 2a), since HPAI H5N1 causes, in contrast to pdmCa09, acute, lethal viral encephalitis in mice.

**Reviewer #3**

In this manuscript, the authors tested a small molecule (4’-fluorouridine , 4’-FIU) as an antiviral for the influenza virus (IV). The work encompasses both in vitro, ex vivo, in vivo work and includes an attempt to assess the mechanism of action.

Some in vitro experiments are rigorously presented, with exceptions noted below.

The ex vivo data in human airway epithelium (HAE) are a good addition but the data presented are incomplete and not adequately described (see below). Additionally, the word “organoids” is misleading and is generally used for different kinds of engineered tissues. This should be corrected (not just the semantics but the actual consideration of what these tissues reflect) or alternatively, the experiment should be conducted on lung tissues.

This is a fair criticism. We have changed the wording throughout in the revised manuscript, now referring to HAE cultures grown at air-liquid interface, to avoid misunderstandings.

The in vivo data are stronger and overall suggest that the 4’-FIU is effective even when administered 24 hours after initial infection. This is in line with the efficacy of the neuraminidase inhibitors currently approved and in use for IV. The authors claim that the advantage of their strategy is the high barrier to resistance, but no data are presented in this regard. The authors should either present data on the viral evolution of their small molecule compared to the other available antivirals to substantiate their claim or remove this claim.

Neuraminidase inhibitors such as oseltamivir phosphate are actually reportedly inactive against pdm09 H1N1 in animal models (i.e. PMID 31645453 and reference 45 of our manuscript). In contrast, 4’-FlU significantly reduced lung virus load and mediates complete survival animals when treatment is initiated up to 60 hours after infection with pdm09 H1N1, which to our knowledge is highly unusual if not unprecedented. We do agree, however, that based on the data presented one cannot conclude that barrier to resistance is high. Please see in this context our response to comment #2 of reviewer #1. This comment was based on the exciting results of our evolving resistance profiling study, but have removed references to the possible height of the resistance barrier from this revised manuscript, since we will address this topic comprehensively in a designated publication.

The 4’-FIU small molecule has been shown to have broad-spectrum activity vs. other viruses like SARS-CoV-2 and RSV. For this reason, the novelty of the work is modest.

We believe that the 2021-2023 H5N1 HPAI epidemic, the largest on record, best illustrates why it is imperative to diversify the therapeutic arsenal available against influenza through the development of next-generation antivirals. Broad-spectrum orally available drugs are in our opinion the most promising strategy to serve as a first-line defense against emerging or re-emerging viral pathogens. We have indeed previously reported activity of 4’-FlU against SARS-CoV-2 and RSV. However, it is unclear to us how one can jump from this information to the conclusion that the compound is exquisitely potent against seasonal, pandemic, and highly pathogenic avian influenza viruses, blocks IAV transmission, has an unusually wide therapeutic time window for treatment of IAV infection *in vivo*, and inhibits influenza virus polymerase through a distinct MOA (immediate chain termination) from that observed for RSV and SARS-CoV-2 polymerase (delayed and sequence-context dependent delayed chain termination, respectively). We consider these novel findings to be of very high impact, since they pave the path towards formal development of 4’-FlU for the treatment of influenza.

Specific comments:
-Fig 1b presents data from in vitro infection with several strains however no details of the experiment are presented.

We have expanded the Methods section in this revision, now providing specific experimental detail for the dose-response experiments shown in figure 1b (page 17, lines 410-426).

-Fig 1b shows a wide range of efficacy with EC90 varying from 0.06 to 5.347uM. The authors should comment on these differences. Is the difference due to the inherent partial resistance of the different strains? Since the authors have an assay to assess the activity (Fig.1d to f), the molecular underpinnings for the difference in activity should be tested. Otherwise, concerns are raised about the lack of understanding of the basis for these differences.

Please see our above response to comment #2 of reviewer #1. Identifying and validating resistance mutations, rebuilding confirmed mutations in RdRP expression constructs, expressing and purifying the corresponding mutant polymerase complexes, and evaluating the effect on activity in the *in vitro* assay is feasible, but constitutes a major undertaking. Resistance profiling of 4’-FlU is at an advanced stage, albeit still ongoing, and includes in addition to the biochemical work-up also an assessment of the implication of resistance on viral pathogenicity and retained *in vivo* 4’-FlU efficacy. As outlined above, we have clarified in this revision that resistance profiling will be required to explore the molecular basis for these strain-specific differences in sensitivity to the compound (page 13, lines 318-323), but decided against trying to force a *bona fide* full-length resistance profiling manuscript into the present article.

-Fig 1c is missing crucial details about the experiment (time points, viral dosage, etc.). Since the mouse-adapted influenza virus is self-extinguishing in this tissue and human strains totally destroy the tissue, information about the TEER and images of the infected HAE during the course of infection should be provided to evaluate the protective effect of the 4’-FIU over time.

Influenza virus infects predominantly ciliated cells in this model. Whereas these cells are ultimately destroyed, barrier function of the infected epithelium model is reportedly maintained, resulting in flat TEER values (PMID 28004801). We have added our TEER measurements, which confirm this observation, as a new subpanel (S1c Fig) to supplementary figure 1 and refer to PMID 28004801 in the text (page 6, lines 109-113). We have not accompanied this experiment with confocal imaging, however, since we have demonstrated low toxicity and efficient anabolism of 4-FlU to the corresponding triphosphate form in HAE air-liquid interface models extensively in our previous work, which included extensive confocal imaging (PMID 34855509). Repeating, rather than referencing, these previous findings could be perceived as an attempt of double-publication. We therefore prefer not to distract from the overarching focus of the present study of assessing anti-influenza virus efficacy of 4’-FlU *in vivo*. As part of the revision of the Methods section, we have, however, expanded the description of technical detail for this experiment that is now shown as S1b Fig (page 17, line 428 to page 18, line 437).

-Fig 1b and Fig 1c: there is a major difference in activity vs. the same virus (EC90 0.7uM in MDCK and 0.074uM in HAE). In HAE it seems that the potency is much higher than in MDCK. The authors should comment on this difference which points to an underlying mechanism that needs to be explained.

We have reported a comparable potency difference of 4’-FlU between immortal cell lines and HAE cultures for RSV in our previous work (PMID 34855509); in the case of RSV, active concentrations were approximately 17-fold lower in differentiated HAE cultures than immortal cell lines. In this revision, we have commented on this consistent potency difference (page 5, lines 104-106) and discuss two contributing factors: i) more efficient intracellular anabolism of 4’-FlU to the bioactive triphosphate form 4’-FlU-TP in primary cells compared to cell lines, which is not unusual for nucleoside analog inhibitors; and ii) altered viral replication kinetic in differentiated versus undifferentiated HAEs (page 13, line 323 to page 14, line 326). Ultimately, the differentiated HAEs, but not undifferentiated cells, represent a disease-relevant model.

**Editorial Comments**

Thank you very much for submitting your manuscript "4’-Fluorouridine mitigates lethal infection with pandemic human and highly pathogenic avian influenza viruses" for consideration at PLOS Pathogens. As with all papers reviewed by the journal, your manuscript was reviewed by members of the editorial board and by several independent reviewers. The reviewers appreciated the attention to an important topic. They have requested that a number of issues be addressed, including adding statistical analyses, more carefully considering what HAE cultures represent, and discussing several observations in more detail. I ask that you address these concerns as you revise your manuscript. Based on the reviews, we are likely to accept this manuscript for publication, providing that you modify the manuscript according to the review recommendations.

Thank you very much for this evaluation of our work and your editorial guidance.

Please prepare and submit your revised manuscript within 30 days. If you anticipate any delay, please let us know the expected resubmission date by replying to this email.

When you are ready to resubmit, please upload the following:

[1] A letter containing a detailed list of your responses to all review comments, and a description of the changes you have made in the manuscript.

Please note while forming your response, if your article is accepted, you may have the opportunity to make the peer review history publicly available. The record will include editor decision letters (with reviews) and your responses to reviewer comments. If eligible, we will contact you to opt in or out

[2] Two versions of the revised manuscript: one with either highlights or tracked changes denoting where the text has been changed; the other a clean version (uploaded as the manuscript file).

Prepared and uploaded as instructed.

Figure Files:

While revising your submission, please upload your figure files to the Preflight Analysis and Conversion Engine (PACE) digital diagnostic tool, <https://pacev2.apexcovantage.com>. PACE helps ensure that figures meet PLOS requirements. To use PACE, you must first register as a user. Then, login and navigate to the UPLOAD tab, where you will find detailed instructions on how to use the tool. If you encounter any issues or have any questions when using PACE, please email us at [figures@plos.org](mailto:figures@plos.org).

Figures have been converted using PACE.

Data Requirements:

Please note that, as a condition of publication, PLOS' data policy requires that you make available all data used to draw the conclusions outlined in your manuscript. Data must be deposited in an appropriate repository, included within the body of the manuscript, or uploaded as supporting information. This includes all numerical values that were used to generate graphs, histograms etc.. For an example see here: [http://www.plosbiology.org/article/info%3Adoi%2F10.1371%2Fjournal.pbio.1001908#s5](https://nam11.safelinks.protection.outlook.com/?url=http%3A%2F%2Fwww.plosbiology.org%2Farticle%2Finfo%253Adoi%252F10.1371%252Fjournal.pbio.1001908%23s5&data=05%7C01%7Crplemper%40gsu.edu%7Ca00f207e02d245d50a9d08db01648bc5%7C515ad73d8d5e4169895c9789dc742a70%7C0%7C0%7C638105303592748433%7CUnknown%7CTWFpbGZsb3d8eyJWIjoiMC4wLjAwMDAiLCJQIjoiV2luMzIiLCJBTiI6Ik1haWwiLCJXVCI6Mn0%3D%7C3000%7C%7C%7C&sdata=gcjWPgtv2zrVFPNhuoZRJ2knPw5RKalSOqbnEMHQ1co%3D&reserved=0).

All numerical source data and statistical analyses have been provided in the S1 and S2 Data files, respectively.

Reproducibility:

To enhance the reproducibility of your results, we recommend that you deposit your laboratory protocols in protocols.io, where a protocol can be assigned its own identifier (DOI) such that it can be cited independently in the future. Additionally, PLOS ONE offers an option to publish peer-reviewed clinical study protocols. Read more information on sharing protocols at [https://plos.org/protocols?utm_medium=editorial-email&utm_source=authorletters&utm_campaign=protocols](https://nam11.safelinks.protection.outlook.com/?url=https%3A%2F%2Fplos.org%2Fprotocols%3Futm_medium%3Deditorial-email%26utm_source%3Dauthorletters%26utm_campaign%3Dprotocols&data=05%7C01%7Crplemper%40gsu.edu%7Ca00f207e02d245d50a9d08db01648bc5%7C515ad73d8d5e4169895c9789dc742a70%7C0%7C0%7C638105303592748433%7CUnknown%7CTWFpbGZsb3d8eyJWIjoiMC4wLjAwMDAiLCJQIjoiV2luMzIiLCJBTiI6Ik1haWwiLCJXVCI6Mn0%3D%7C3000%7C%7C%7C&sdata=B5E06GgjoMidSqmHMTMyEsZtD1hY4x7ypAX8FjIg5Ew%3D&reserved=0)

Detailed methods have been provided in the revised Methods section.

References:

Please review your reference list to ensure that it is complete and correct. If you have cited papers that have been retracted, please include the rationale for doing so in the manuscript text, or remove these references and replace them with relevant current references. Any changes to the reference list should be mentioned in the rebuttal letter that accompanies your revised manuscript. If you need to cite a retracted article, indicate the article’s retracted status in the References list and also include a citation and full reference for the retraction notice.

None of the papers cited have been retracted. We have added one reference (new reference 27) in response to a reviewer question as specified above (response to question of reviewer #3 concerning Fig 1c).
